# Comparative proteomic analysis of papaya bud flowers reveals metabolic signatures and pathways driving hermaphrodite development

**DOI:** 10.1038/s41598-024-59306-x

**Published:** 2024-04-17

**Authors:** Rafaela Pereira Duarte, Helaine Christine Cancela Ramos, Lucas Rodrigues Xavier, Adriana Azevedo Vimercati Pirovani, Alex Souza Rodrigues, Dayana Kelly Turquetti-Moraes, Izaias Rodrigues da Silva Junior, Thiago Motta Venâncio, Vanildo Silveira, Messias Gonzaga Pereira

**Affiliations:** 1 Laboratório de Melhoramento Genético Vegetal – LMGV, Universidade Estadual do Norte Fluminense Darcy Ribeiro-UENF, Campos dos Goytacazes, 28.013-602 Brazil; 2 Laboratório de Biotecnologia – LBT, Universidade Estadual do Norte Fluminense Darcy Ribeiro-UENF, Campos dos Goytacazes, 28.013-602 Brazil; 3 Unidade de Biologia Integrativa, Setor de Genômica e Proteômica, Universidade Estadual do Norte Fluminense Darcy Ribeiro-UENF, Campos dos Goytacazes, 28.013-602 Brazil; 4 Laboratório de Química e Função de Proteínas e Peptídeos - LQFPP, Universidade Estadual do Norte Fluminense Darcy Ribeiro-UENF, Campos dos Goytacazes, 28.013-602 Brazil

**Keywords:** Papaya, Proteome, Sexual differentiation, Floral development, Proteomics, Agricultural genetics

## Abstract

Papaya (*Carica papaya*) is a trioecious species with female, male, and hermaphrodite plants. Given the sex segregation, selecting hermaphroditic plants is vital for orchard establishment due to their greater commercial value. However, selecting hermaphrodite plants through sexing is laborious and costly. Moreover, environmental stressors can exacerbate the issue by potentially inducing abnormal flower development, thus affecting fruit quality. Despite these challenges, the molecular mechanisms governing sex development in papaya remain poorly understood. Thus, this study aimed to identify proteins associated with sex development in female and hermaphrodite flowers of papaya through comparative proteomic analysis. Proteins from flower buds at the early and late developmental stages of three papaya genotypes (UENF-CALIMAN 01, JS12, and Sunrise Solo 72/12) were studied via proteomic analysis via the combination of the shotgun method and nanoESI-HDMS^E^ technology. In buds at an early stage of development, 496 (35.9%) proteins exhibited significantly different abundances between sexes for the SS72/12 genotype, 139 (10%) for the JS12 genotype, and 165 (11.9%) for the UC-01 genotype. At the final stage of development, there were 181 (13.5%) for SS72/12, 113 (8.4%) for JS12, and 125 (9.1%) for UC-01. The large group of differentially accumulated proteins (DAPs) between the sexes was related to metabolism, as shown by the observation of only the proteins that exhibited the same pattern of accumulation in the three genotypes. Specifically, carbohydrate metabolism proteins were up-regulated in hermaphrodite flower buds early in development, while those linked to monosaccharide and amino acid metabolism increased during late development. Enrichment of sporopollenin and phenylpropanoid biosynthesis pathways characterizes hermaphrodite samples across developmental stages, with predicted protein interactions highlighting the crucial role of phenylpropanoids in sporopollenin biosynthesis for pollen wall formation. Most of the DAPs played key roles in pectin, cellulose, and lignin synthesis and were essential for cell wall formation and male flower structure development, notably in the pollen coat. These findings suggest that hermaphrodite flowers require more energy for development, likely due to complex pollen wall formation. Overall, these insights illuminate the molecular mechanisms of papaya floral development, revealing complex regulatory networks and energetic demands in the formation of male reproductive structures.

## Introduction

Most angiosperms are hermaphrodite plants, with only 5% of plants being monoecious and 6% being dioecious^1^. Unisexual plants are derived from hermaphrodite ancestors as a result of an adaptive process that allows an increase in genetic variation through crossing. Two possible routes have been proposed for the evolution of dioecious plants: via monoecious or gynodioecious plants^2^. Therefore, unisexual flowers arise from the suppression of the development of one of the sexual organs. In type I unisexual flowers, this process occurs at the stage of cellular specification of the floral meristem, which leads to the non-production of one of the sexual organs (stamen or carpel). Type II unisexual flowers develop late, with a vestige of the opposite sex organ present^3^.

Papaya (*Carica papaya*) is a trioecious plant, which includes female, male, and hermaphrodite plants. Natural papaya populations are dioecious (female and male plants), while cultivated populations are gynoecious andromonoecious (female and hermaphrodite plants). Male papaya flowers are type II, with traces of an evident carpel, while female flowers (type I) have no traces of stamens^4^. These findings reinforce the preliminary evidence suggesting that trioecious plants are derived from a dioecious ancestor through the loss of carpel suppressive function^2^.

Sex determination in papaya was initially proposed to be controlled by a single gene with three alleles^5^. Later, high-density genetic mapping and physical mapping revealed a sex determination locus with severe suppression of recombination but a high percentage of polymorphic markers. This discovery provides direct evidence supporting the origin of recently evolved sex chromosomes (XX female, XY male, and XY^h^ hermaphrodite), with a genomic region on the Y and Y^h^ chromosomes in which recombination with X is suppressed^6,7^. Analysis of sequence divergence between the four X and Y^h^ gene pairs revealed an estimated age of divergence between 0.5 and 2.2 million years, a timeframe considered recent in evolutionary history^8^.

The sex-determining region of the sex chromosomes is short and well characterized; the hermaphrodite and male-specific regions (HSY and MSY) of the Y^h^ and Y chromosomes correspond to 8.1 Mb (15% of chromosome 1), and the female region of the X chromosome corresponds to 3.5 Mb (6% of chromosome 1). The Y and Y^h^ chromosomes are nearly identical, with only 0.4% divergence in their sex-specific regions^9,10^. The HSY region, its X-chromosome and its X-chromosome counterpart have 121 genes, 56 of which are HSY-specific and 74 of which are X specific. However, the gene content, the structure of the exons, and the order of the genes between the entire HSY and MSY are conserved^10,11^.

Sexual differentiation has a significant impact on the papaya crop, as only hermaphrodite plants yield commercially valuable fruits. To ensure the orchards of hermaphrodite plants, three to four papaya seedlings are typically planted per hole, with female plants thinned out post-sexing. However, this practice not only hampers plant development but also results in wasted cultivation areas and increased resource consumption. Molecular markers have already been developed for sexing young papaya seedlings, but molecular analyses still incur significant costs, and leaf tissue must be collected from all plants in the field or in a greenhouse. Moreover, in addition to the costly sexing process, environmental stresses can lead to abnormal flower development in hermaphrodite plants (female sterility, pentandry and carpelloidy), impacting fruit development.

Understanding sexual differentiation in papaya plants remains challenging owing to their intricate nature. MADS-box genes are pivotal regulators of floral organ identity, and some likely play crucial roles in sex determination in papaya. Notably, the sex-related MADS-box genes CpSTK and CpSEP3 exhibit female-biased expression^12,13^, while Cp2671 is uniquely found on the Yh and Y chromosomes but not on the X chromosome^14^. While several other MADS-box genes have been identified in papaya, most seem to have indirect effects on sex determination rather than serving as causative agents^12,15^.

The process of sex differentiation in papaya appears to be governed by a multitude of regulatory and coordinating factors, including transcriptional, epigenetic, and phytohormonal mechanisms. Comparative transcriptome analysis between males and females revealed 11 genes within the sex determination region that exhibited significant differential expression, including transcription factor genes and genes involved in methylation or chromatin structure modification. In addition to genes within the sex determination region, hormones—particularly ABA and auxin—and other transcription factors are thought to play roles in the sex differentiation process in papaya^16^. Other studies have highlighted that DNA methylation is also a significant player in sexual differentiation and sex chromosome evolution. Whole-genome bisulfite sequencing of papaya early-stage flowers revealed that distinct phytohormone signaling pathways and differential methylation-related gene expression are thought to contribute to DNA methylation alterations in papaya^17^.

Proteomics has been widely employed in plant science as a powerful tool for protein identification and analysis of gene function. Comparative proteomics stands out within other subareas, and its objective is not to identify the entire set of proteins in a specific sample but rather to establish differences in protein profiles between different groups, such as genotypes, cell types, tissues, organs, and stages of development^18,19^. Comparative proteomics analyses of the flower buds of dioicous species have provided intriguing insights into the molecular intricacies governing sexual dimorphism in flower development. In kiwifruit (*Actinidia chinensis* var. *chinensis*), notable disparities were detected in catalytic activity-related proteins, with significant involvement of the plant hormone signaling pathway in distinguishing male and female flowers, as well as variations in flavor biosynthesis, phyllopanoid biosynthesis, and sucrose metabolism^20^. Conversely, *Coccinia grandis* exhibited conserved ethylene-mediated stamen inhibition and exhibited male-biased expression of proteins pivotal for pollen germination and tube growth^21^. In *Pistacia chinensis*, differential protein expression was observed for oxidative stress resistance and photosynthetic pathways during female and male primordium differentiation, indicating seasonal variations^22^.

However, no proteomic studies have sought to identify proteins involved in the process of sexual differentiation in papaya. Given the pivotal role of floral development and sexual differentiation in optimizing fruit production, the intricate processes governing these aspects in papaya demand further exploration. Therefore, this study endeavored to fill this gap by employing a shotgun comparative proteomics approach in hermaphrodite and female papaya flowers. The aim was to unravel the intricate molecular mechanisms underlying sex differentiation, shedding light on crucial factors influencing sexual development in this species.

## Material and methods

### Plant material

Proteomic analyses were subsequently conducted with the hybrid UENF-CALIMAN 01 (UC-01) and its parents JS12 and Sunrise Solo 72/12. The UC-01 hybrid, the first national hybrid, was developed by the Universidade Estadual do Norte Fluminense Darcy Ribeiro and the company Caliman Agrícola S/A for the North and Northwest regions of Rio de Janeiro and the northern portion of the coast of Espírito Santo. The UC-01 genotype has the phenotypic characteristics of the Formosa group, which consists of a vigorous plant with high health and high productive potential^23^. The parental genotypes were selected by the pedigree method and belong to the papaya breeding program of the UENF/Caliman germplasm bank^24,25^.

Seeds of each genotype were germinated in a greenhouse in a plastic tray with a capacity of 96 tubes using the commercial substrate Basaplant^®^. A total of 160 seedlings (60 from each lineage and 40 from the hybrid) were transplanted to the experimental area belonging to the Colégio Agrícola Antônio Sarlo, located in Campos dos Goytacazes/RJ. The spacing between rows was 1.0 m, and that between plants was 1.0 m. Five months post-transplantation, flower buds were harvested at both the early (7 mm in length) and late (20 mm in length) developmental stages following the classification and length criteria outlined by Urasaki et al.^14^. The methodology involved a transcriptome analysis of flower samples collected from male, female, and hermaphrodite plants, employing high-throughput SuperSAGE technology for digital gene expression analysis^14^. Subsequently, the samples were frozen in liquid nitrogen and preserved at − 80 °C until protein extraction.

### Total protein extraction

The experiment consisted of 12 different biological samples: two sexes (female and hermaphrodite) × two developmental stages (late and early) × three genotypes (UC-01, JS12, and SS72/12). Analyses were performed using three technical replicates with each biological A total of 300 mg of fresh matter was used for each obtained biological sample, which was macerated in liquid nitrogen using a mortar and ceramic crucible. The pulverized material was transferred to microtubes and resuspended in 1 mL of extraction buffer [10% trichloroacetic acid (TCA; Sigma‒Aldrich) in acetone].

The samples were incubated for 60 min at 4 °C and then centrifuged at 16,000 × g for 30 min. The supernatant was discarded, and the pellet was washed three times in cold acetone with 20 mM DTT. Subsequently, the samples were resuspended in 1 mL of buffer (7 M urea, 2 M thiourea, 2% Triton X-100, 1% DTT (DTT, GE Healthcare, Piscataway, USA), 1 mM phenylmethanesulfonyl fluoride (PMSF, Sigma‒Aldrich), and a complete cocktail of protease inhibitors (Roche Diagnostics, Mannheim, Germany), vortexed for 5 min, cooled on ice for 30 min, stirred for 30 min at 8 °C and then centrifuged at 16,000 × g for 20 min at 4 °C. The supernatants were collected and stored at − 20 °C. Protein quantification was performed using the 2-D Quant Kit (GE Healthcare).

### Protein digestion

Before the trypsin digestion step, 100 µg of protein from each sample was precipitated using the methanol/chloroform method to remove interferents from the samples^26^. The samples were resuspended in a 7 M urea/2 M thiourea solution after protein precipitation. Tryptic protein digestion was performed using the filter-aided sample preparation (FASP) method described by Wiśniewski, et al.^27^, with modifications performed by Burrieza et al.^28^. Before the digestion procedure was started, an integrity test was performed to check for damaged Microcon-30 kDa (Merck Millipore, Darmstadt, HE, Germany) filter units (Hernández-Valladares et al., 2016); thus, only the working units were used. After that, protein aliquots were added to Microcon-30 kDa filter units, washed with 200 µl of 50 mM ammonium bicarbonate (solution A; Sigma-Aldrich), and centrifuged at 10,000 × g for 15 min at 25 °C (unless stated otherwise, all centrifugation steps were performed under these conditions). This step was repeated once for the complete removal of urea before the reduction of proteins. Next, 100 µl of 50 mM DTT, freshly made in solution A, was added, the mixture was gently vortexed, and incubated for 20 min at 60 °C (1 min of agitation and 4 min of rest at 47 g). Then, 200 µl of 8 M urea in 50 mM ammonium bicarbonate (solution B) was added and centrifuged for 15 min. For protein alkylation, 100 µl of 50 mM iodoacetamide (GE Healthcare) freshly prepared in solution B was added, gently vortexed, and incubated for 20 min at 25 °C in the dark (1 min agitation and 19 min resting, at 47 g). Next, 200 µl of solution B was added, and the mixture was centrifuged for 15 min. This step was repeated once. Then, 200 µl of solution A was added, and the mixture was centrifuged for 15 min. This step was repeated twice. Approximately 50 µl of sample should remain in the last wash. For protein digestion, 25 µl of 0.2% (v/v) RapiGest (Waters, Manchester, UK), 25 µl of V5111 trypsin solution (1:100 enzyme:protein; Promega, Madison, WI, USA) were added, and the mixture was gently vortexed and incubated for 18 h at 37 °C (1 min agitation and 4 min resting at 47 g). For peptide elution, the filter units were transferred to new microtubes and centrifuged for 10 min. Then, 50 µl of solution A was added, and the mixture was centrifuged for 15 min. This step was repeated once. For RapiGest precipitation and trypsin inhibition, 5 µl of 15% trichloroacetic acid (TCA; Sigma-Aldrich) was added, the mixture was gently vortexed, and incubated for 30 min at 37 °C. Then, the samples were centrifuged for 15 min, and the supernatants were collected and vacuum dried. Peptides were resuspended in 100 µl of 95% 50 mM ammonium bicarbonate, 5% acetonitrile and 0.1% formic acid (Sigma-Aldrich).

The resulting peptides were quantified using the A205 nm protein and peptide method with a NanoDrop 2000c spectrophotometer (Thermo Fisher Scientific, Waltham, USA). The FASP method efficiently eliminates detergents, salts, and other contaminants that might interfere with spectrophotometric quantification, ensuring that the peptides are in a purified state for accurate analysis.

### Mass spectrometry

Mass spectrometry was performed using a nanoAcquity ultra performance liquid chromatograph (UPLC) coupled to a Q-TOF SYNAPT G2-Si instrument (Waters). Three biological replicates of 2 µg of digested protein were run. The samples were loaded during separation onto a 5-µm nanoAcquity UPLC M-Class Symmetry C18 trap column (180 µm × 20 mm) at 5 µL min^−1^ for 3 min and then onto a 1.8 µm (75 µm × 150 mm) nanoAcquity M-Class HSS T3 reversed-phase column at 400 nL min^−1^, with a column temperature of 45 °C.

A binary gradient was used for peptide elution. Phase A consisted of water (Tedia, Fairfield, Ohio, USA) and 0.1% formic acid (Sigma-Aldrich), and phase B consisted of acetonitrile (Sigma‒Aldrich) and 0.1% formic acid (Sigma‒Aldrich). Gradient elution started with B 5%, which increased from B 5 at 40% for up to 91.12 min and B 40 at 99% for up to 95.12 min, after which it was maintained at 99% for up to 99.12 min. Then, the elution decreased to B 5% until 101.12 min and maintained at B 5% until the end of the run at 117.00 min. Analyses were performed in positive, resolution mode (V mode) at 35,000 FWHM with ion mobility (HDMSE), and data-independent acquisition (DIA) mode with the ion mobility wave velocity program starting at 800 m s^−1^ and ending at 500 m s^−1^. Collision energy transfer increased from 25 to 55 V in high energy mode, the cone and capillary voltages were 40 and 2800 V, respectively, the nanoflow gas pressure was 0.5 bar, the flow rate of the purge gas was 150 L h^−1^, and the source temperature was 100 °C. The sweep time was set to 0.6 s in continuous mode for the time-of-flight (TOF) parameters, with a mass range of 50–2000 Da. Human [Glu1]-fibrinopeptide B (Sigma-Aldrich) at 100 fmol μL^−1^ was used as an external calibrator, and lock mass acquisition was performed every 30 s. Mass spectra were subsequently acquired by MassLynx v4.1 software (Waters).

### Data analysis

The processing of the spectra and reference bank was performed using ProteinLynx Global SERVER (PLGS) v.3.02 (Waters) software, and the workflow was obtained with ISOQuant software^29,30^. The following parameters were used in the PLGS analysis: Apex3D of 150 counts as low energy limit, 50 counts as high energy limit, and 750 counts as intensity limit; a lost cleavage; at least three ion fragments per peptide; at least seven ion fragments per protein; at least two peptides per protein; fixed modifications such as carbamidomethyl (C) and variable modifications such as oxidation (M) and phosphoryl (STY); and a false discovery rate (FDR) for peptide and protein identification, which was adjusted to a maximum of 1%, with a minimum length of six amino acids.

The Phytozome 10.2 database (https://phytozome.jgi.doe.gov/pz/portal.html), containing all *C. papaya* protein entries (27,793 sequences, July 2021), was used. Quantification was performed by ISOQuant v.1.7 software using previously described settings and algorithms^29,30^. Multidimensional normalization, which corrects the peak intensities based on the intensity and retention time domains, was used. In brief, the analysis included alignment of the retention time and the exact mass retention time (EMRT). Quantitative label-free analyses were estimated using the TOP3 quantification approach^31^, followed by the multidimensional normalization process implemented in ISOQuant^29^. Only proteins present in three technical replicates were subjected to differential abundance analysis after the data were processed to ensure the quality of the results. Finally, the proteins were subjected to functional characterization using OmicsBox software (www.biobam.com/omicsbox/). The sequences associated with biological processes not identified by OmicsBox were manually analyzed using the following online BLAST tools: UniprotKB (http://www.uniprot.org/blast/), NCBI (http://www.ncbi.nlm. nih.gov), and Phytozome (https://phytozome.jgi.doe.gov/pz/portal.html).

The genome of the SunUp cultivar, deposited in 2021 at the National Genomics Data Center (NGDC), whose assembly is at the chromosomal level, was used as a reference for chromosomal mapping of the genes referring to the proteins identified in the present study^11^.

### Comparative proteomic analysis

The analysis included comparisons of hermaphrodite flower buds relative to female flower buds from the three genotypes analyzed in the two phases of floral development. After ISOQuant data analyses, only the proteins that were either present or absent (for unique proteins) in all three biological replicates were considered for differential comparative analysis. The data were analyzed using Student’s t test (two-tailed). Proteins with *P*-values of *P* < 0.05 were considered differentially accumulated proteins (DAPs) if the Log_2_ of the fold change (Log_2_ FC) was greater than 0.5 (up-regulated) or if the Log_2_ FC was lower than − 0.5 (down-regulated). The enrichment of biological processes, cellular components, molecular functions, and KEGG pathways between DAPs was assessed through the Metascape portal^30^ after a BLAST at NCBI (https://www.ncbi.nlm.nih.gov) was used to obtain the sequences of reference proteins in *A. thaliana*. Fisher’s exact test (*p* < 0.01) was used for the analysis.

## Results

### *Carica papaya* flower bud proteome

Comparative proteomic analysis of hermaphrodite and female flower buds was carried out at two stages of development and for three papaya genotypes. A total of 1402 and 1352 proteins were identified in the early and late stages of development, respectively (Supplementary Tables 1 and 2).

**Figure 1:**
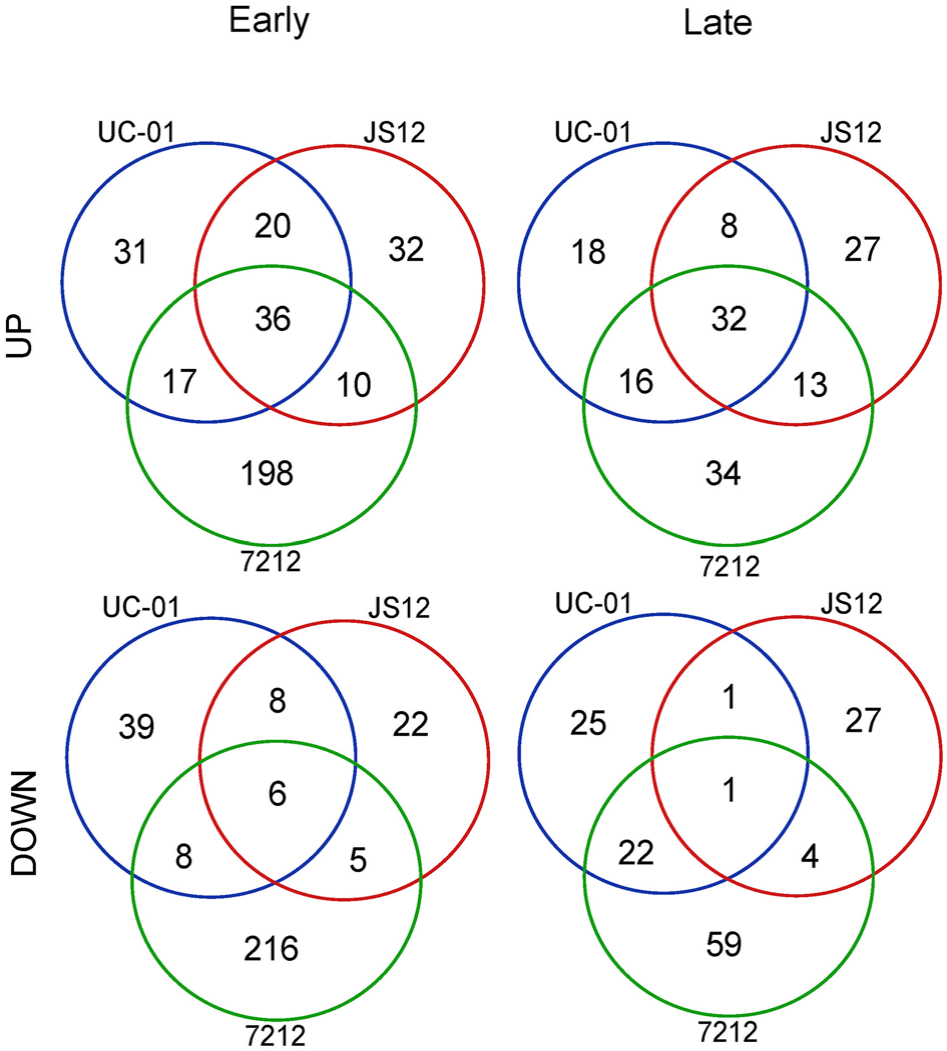
Venn diagram of DAPs in hermaphrodite and female flower buds of three papaya genotypes (UC-01, JS12, and SS72/12) at two stages of development (early and late).

According to the differential proteomics analysis between hermaphrodite flower buds and female buds at an early stage of development, 496 (35.9%) proteins were differentially regulated (*P* < 0.05, |Log_2_ FC|> 0.5) for the SS72/12 genotype, 139 (10%) for the JS12 genotype, and 165 (11.9%) for the UC-01 genotype. Among the DAPs, the JS12 and SS72/12 genotypes presented most of their proteins as up-regulated. On the other hand, in addition to the greater amount of DAPs identified in the SS72/12 genotype, a balanced result was observed between the up- and down-regulated proteins (Table 1). Comparing the three genotypes, 36 up- proteins and six down-regulated proteins coincided in the three analyzed genotypes (Fig. 1).

**Table 1:** Number of proteins identified via comparative proteomics analysis (XY^h^/XX) between flower buds at the early and late developmental stages of the three papaya genotypes.

Genotype	Early stage			Late stage		
	7212	JS12	UC-01	7212	JS12	UC-01
Total proteins	1381	1383	1386	1340	1339	1345
Up proteins (XY^h^/XX)	243	89	90	83	62	61
Down proteins (XY^h^/XX)	228	39	55	81	31	46
Unique proteins XY^h^	18	9	14	14	18	13
Unique proteins XX	7	2	6	3	2	3

According to the differential proteomics analysis between hermaphrodite flower buds and female buds at the final stage of development, 181 (13.5%) proteins were differentially regulated for the SS72/12 genotype, 113 (8.4%) for the JS12 genotype, and 125 (9.1%) for the UC-01 genotype. As in the early stage of bud development, most of the DAPs were up-regulated in the JS12 and UC-01 genotypes. Similarly, for the SS72/12 genotype, the numbers of up- and down-regulated proteins were similar (Table 1). A comparison of the three genotypes revealed that 32 up-regulated proteins and one down-regulated protein were associated with the three analyzed genotypes (Fig. 1).

**Figure 2:**
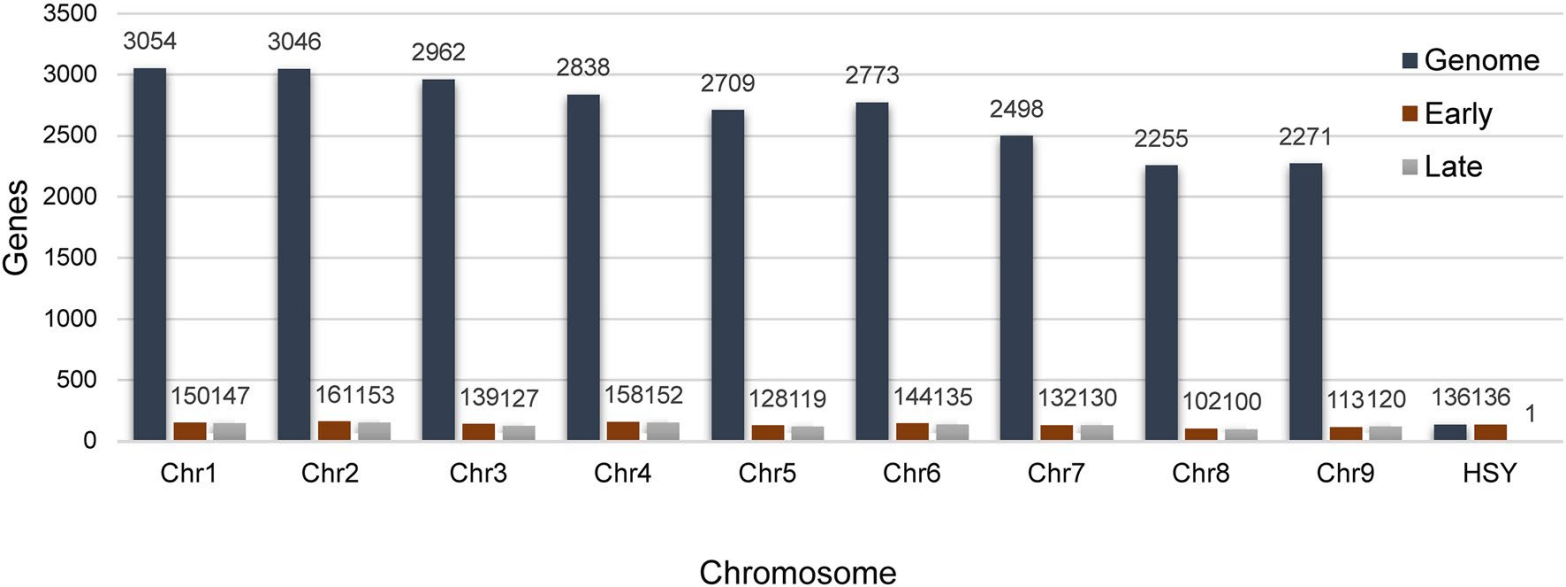
Number of genes for each chromosome. The total number of genes for each of the *C. papaya* chromosomes is shown in dark blue. The numbers of coding genes identified in this analysis are represented in brown and gray, corresponding to the coding regions of the peptides identified by proteomics at the early and late stages of development, respectively. Chr: chromosome.

Chromosomal mapping was also conducted to determine whether the identified proteins were encoded by genes from the sex-specific region of papaya. The genome of the SunUp cultivar, whose assembly was at the chromosomal level, was used as a reference^11^. Among the proteomic accessions, 87.5% were significantly aligned with proteins encoded by gene regions of the genome for both stages of development. Figure 2 shows the number of coding genes for the proteomic accessions on each chromosome.

Only the gene coding for the ARF-GTPase protein was aligned with the HSY region of chromosome 1. However, this protein was not considered DAP according to the criteria analyses. The remaining coding genes were all aligned on autosomal chromosomes or in the autosomal region of chromosome 1.

### Functional annotation of DAPs

GO and KEGG enrichment analyses were also conducted to provide comprehensive insights into the functional roles and regulatory pathways associated with the DAPs in papaya flower buds at different developmental stages and across different genotypes. When examining the differences across genotypes, it is evident that while certain pathways may show consistency in their enrichment across different genotypes, there are also notable variations.

### GO enrichment analysis of flower buds at early stages of development

**Figure 3:**
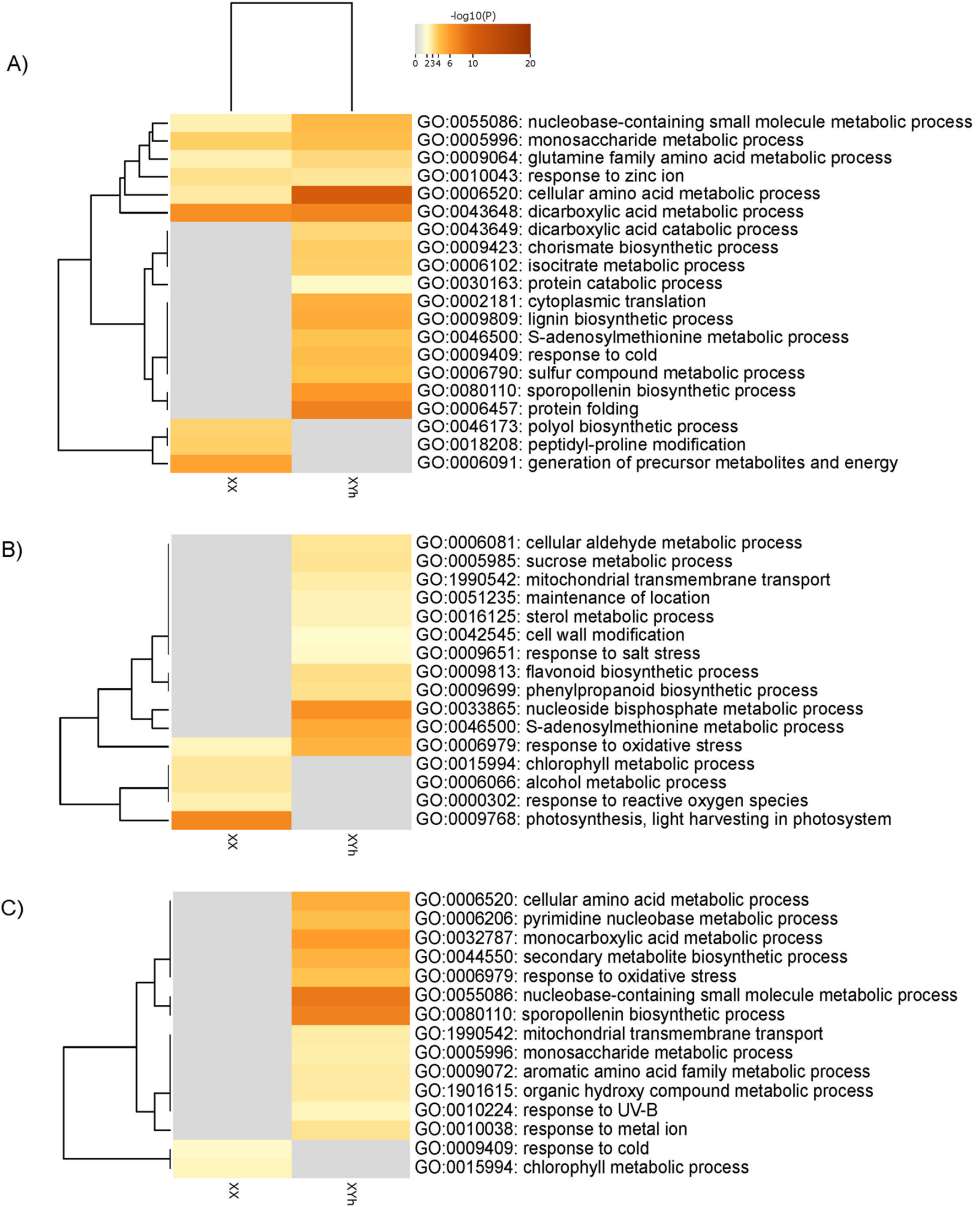
Heatmap of the 20 gene ontology (GO) terms enriched within the “Biological Process” domain that varied the most between the DAPs of hermaphrodite and female flower buds of the (**A**) SS72/12, (**B**) JS12, and (**C**) UC-01 genotypes at the early stage of development (Fisher’s test at *p* < 0.01).

In terms of up-regulated proteins, several biological processes were associated with more than one genotype, suggesting that common molecular processes occur across different papaya genotypes. Specifically, sporopollenin biosynthesis and the response to oxidative stress were enriched in both the SS72/12 and UC-01 genotypes. Additionally, S-adenosylmethionine metabolism is another pathway that occurs in both the SS72/12 and JS12 genotypes. Moreover, up-regulated proteins enriched specifically in the SS72/12 genotype were involved in amino acid metabolism, dicarboxylic acid metabolism, lignin biosynthesis, and protein translation and folding, highlighting genotype-specific molecular processes. Conversely, the JS12 genotype exhibited enrichment in the metabolism of nucleoside biphosphate and biosynthesis of phenylpropanoid, while the UC-01 genotype was characterized by pathways involving nucleotide metabolism and biosynthesis of secondary metabolites (Fig. 3).

A lower number of down-regulated proteins were enriched in the biological process category, indicating fewer common pathways among the genotypes. Only chlorophyll metabolism was common to both the JS12 and UC-01 genotypes. In the SS72/12 genotype, down-regulated proteins are involved in dicarboxylic acid metabolism, precursor metabolite generation and energy generation, amino acid metabolism, carboxylic acid metabolism, and peptidyl-proline modification. The following pathways stand out for the JS12 genotype: photosynthesis, response to reactive oxygen species (ROS), and alcohol metabolism. Finally, cold response pathways were enriched in the UC-01 genotype (Fig. 3).

### GO enrichment analysis of flower buds at the final stage of development

**Figure 4:**
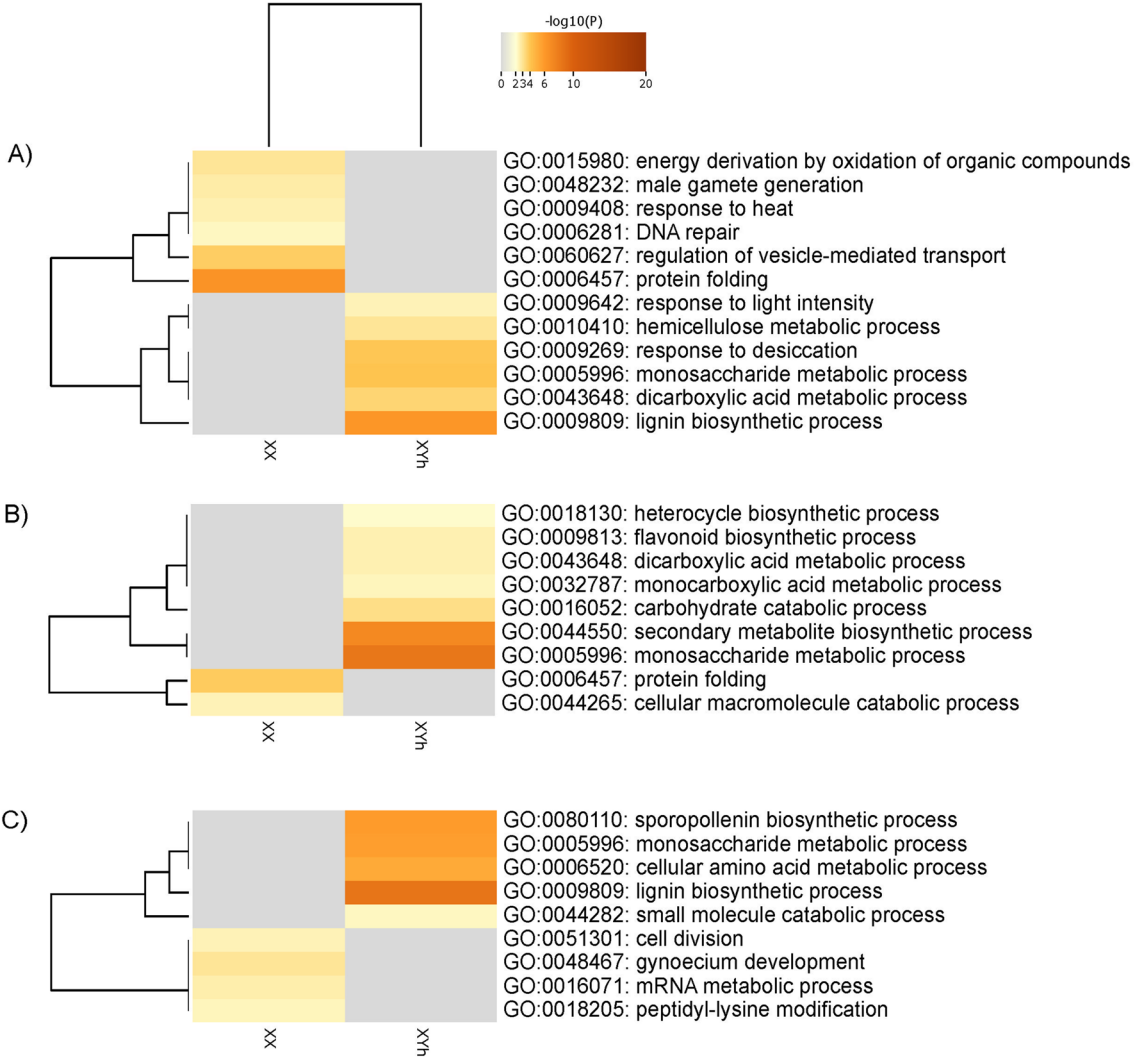
Heatmap of the 20 gene ontology (GO) terms enriched within the “Biological Process” domain that varied the most between the DAPs of hermaphrodite and female flower buds of the (**A**) SS72/12, (**B**) JS12, and (**C**) UC-01 genotypes at the late stage of development (Fisher’s test at *p* < 0.01).

At the final stage of development, up-regulated proteins were enriched in monosaccharide metabolism, which was identified as a common pathway across all three genotypes, indicating its significance in papaya flower maturation. Additionally, lignin biosynthesis occurred in both the SS72/12 and UC-01 genotypes. In the SS72/12 genotype, in addition to monosaccharide metabolism and lignin biosynthesis, pathways related to dissection response, dicarboxylic acid metabolism, hemicellulose metabolism, and response to light intensity were enriched. Conversely, the JS12 genotype showed notable enrichment in secondary metabolite biosynthesis, carbohydrate metabolism, carboxylic acid metabolism, and flavonoid biosynthesis. Similarly, the UC-01 genotype exhibited enrichment in cellular amino acid metabolism (Fig. 4).

Regarding down-regulated proteins, the process of protein folding was enriched in both the SS72/12 and JS12 genotypes. In addition to protein folding, specific DAPs in the SS72/12 genotype are associated with the regulation of vesicle-mediated transport, DNA repair, heat response, and energy derivation by the oxidation of organic compounds. Conversely, the JS12 genotype showed enrichment primarily in the catabolism of cellular macromolecules. Furthermore, pathways related to gynoecium development, mRNA metabolism, peptidyl-lysine modification, and cell division were notably enriched in the UC-01 genotype (Fig. 4).

### KEGG pathway enrichment analysis in flower buds at early stages of development

**Figure 5:**
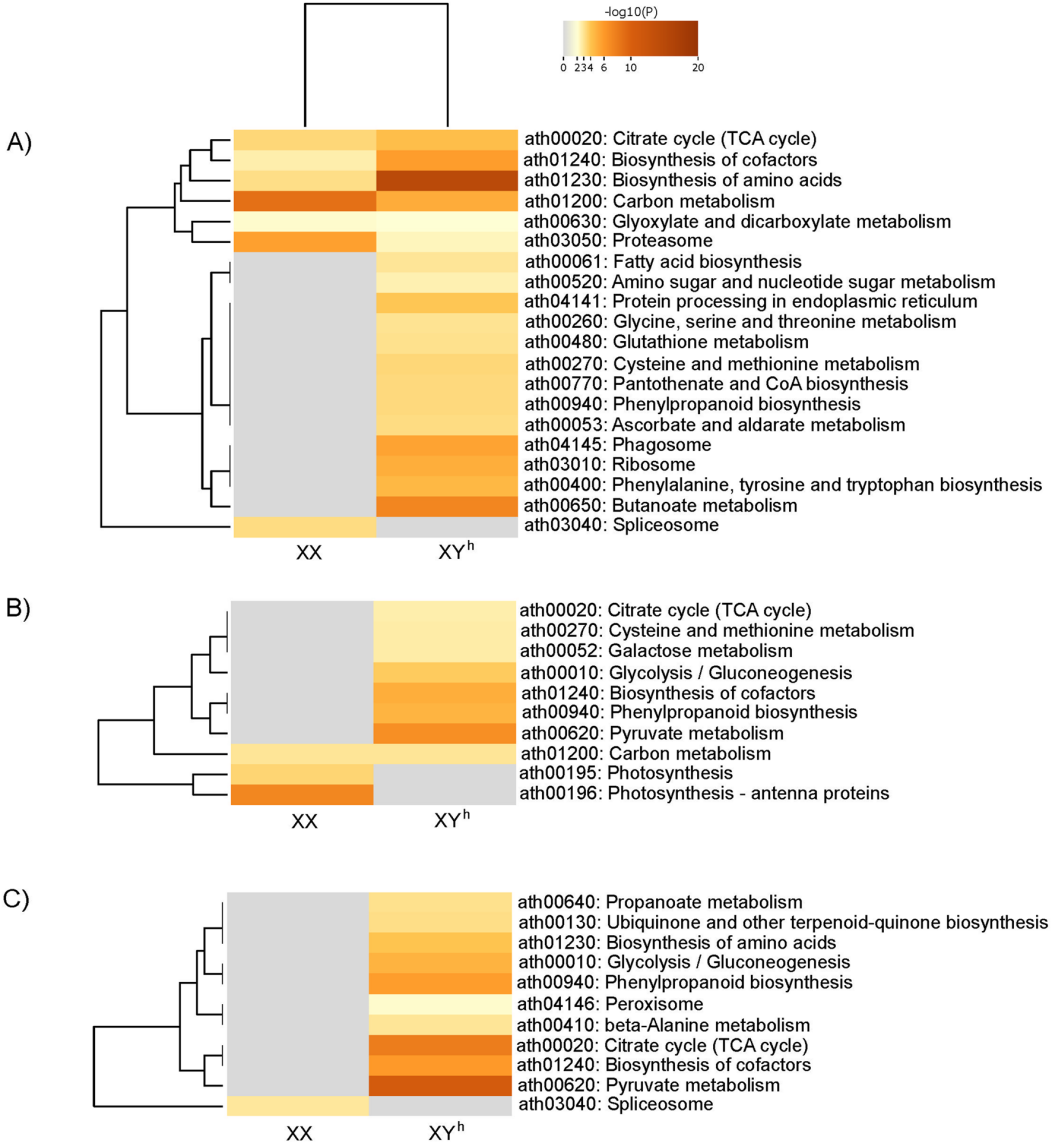
Heatmap of the 20 enriched KEGG pathways that varied the most between the DAPs of hermaphrodite and female flower buds of the (**A**) SS72/12, (**B**) JS12, and (**C**) UC-01 genotypes at the early stage of development (Fisher’s test at *p* < 0.01).

In contrast to the common results observed in the biological process category, the KEGG pathway analysis highlights common metabolic activities across all the genotypes in terms of up-regulated proteins. Key pathways such as carbohydrate metabolism, phenylpropanoid biosynthesis, amino acid metabolism, and cofactor biosynthesis were uniformly enriched. However, notable distinctions emerge upon closer examination. In the SS72/12 genotype, additional enrichment was evident in pathways such as protein translation (ribosome) and transport and catabolism (phagosome) cellular processes, suggesting a broader metabolic landscape (Fig. 5). Similarly, pyruvate metabolism was shown to be a pathway shared between the JS12 and UC-10 genotypes, underscoring common metabolic features. These insights shed light on both the shared and divergent metabolic adaptations among papaya genotypes, offering a deeper understanding of their physiological intricacies.

Among the down-regulated proteins, the pathway involved in carbon metabolism was enriched in both the SS72/12 and JS12 genotypes. Furthermore, protein degradation (via the proteasome) is a pathway that occurs in both the SS72/12 and UC-01 genotypes. Specifically, the SS72/12 genotype exhibited enrichment in transcription (spliceosome), while the JS12 genotype was characterized by enrichment in photosynthesis (Fig. 5).

### KEGG pathway enrichment analysis in flower buds at the final stage of development

**Figure 6:**
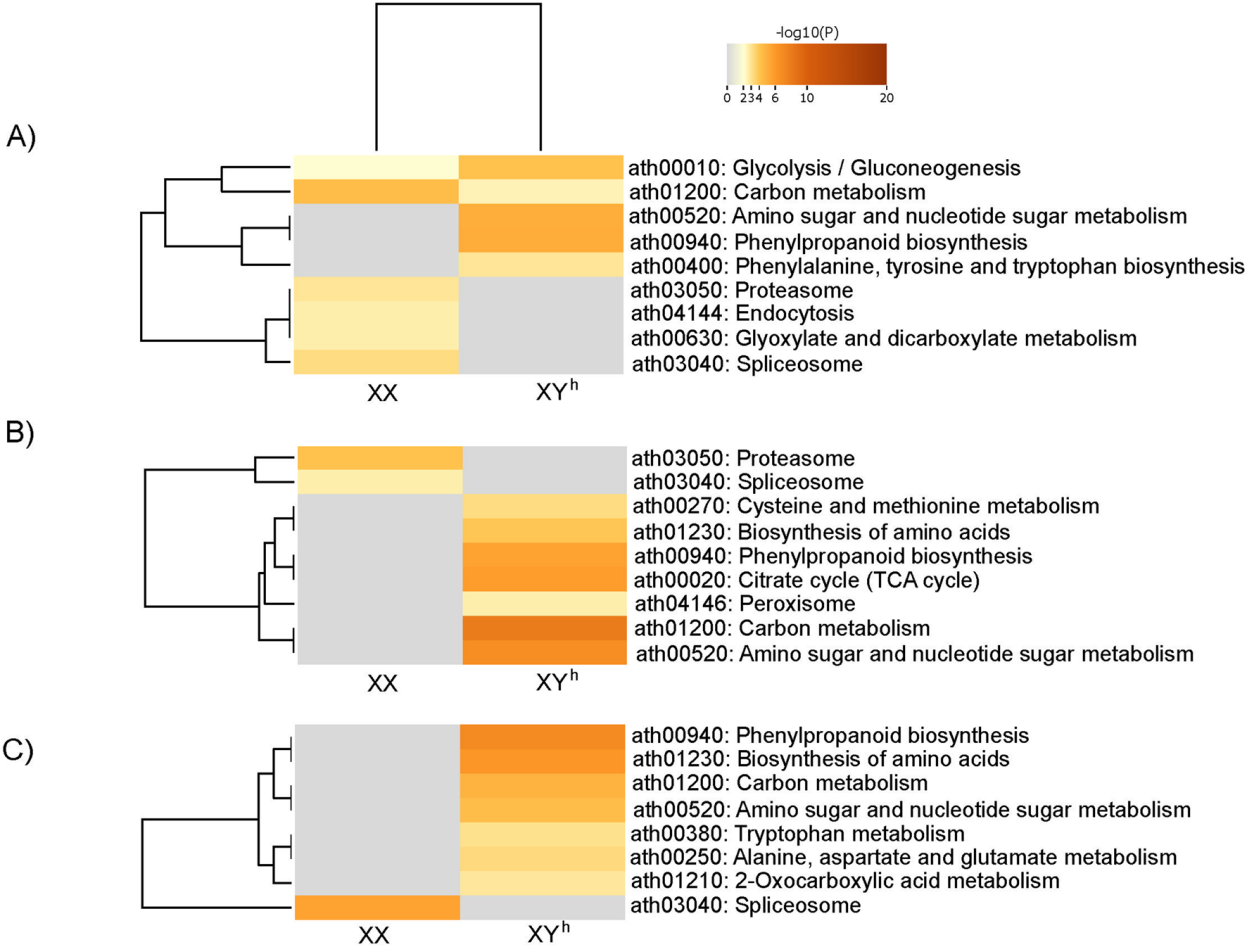
Heatmap of the 20 enriched KEGG pathways that most varied between DAPs of hermaphrodite and female flower buds of the (**A**) SS72/12, (**B**) JS12, and (**C**) UC-01 genotypes at the final stage of development (Fisher’s test at *p* < 0.01).

KEGG pathway enrichment analysis of the up-regulated proteins at the final developmental stage revealed similarities with those at the initial stage, though with a reduced number of pathways identified. Common metabolic activities, such as phenylpropanoid biosynthesis, carbohydrate metabolism, carbon metabolism, amino acid metabolism and biosynthesis, were observed across all the genotypes, indicating consistent molecular processes regardless of the genotype and developmental stage. Furthermore, minimal differences between genotypes suggested a degree of uniformity in metabolic pathways at this developmental stage. Conversely, the enrichment of KEGG pathways associated with down-regulated proteins was notably limited, with only a few pathways identified. Notably, the SS72/12 genotype exhibited a wider array of enriched pathways, including those related to endocytosis, carbon metabolism, and amino acid metabolism, suggesting potential differences in cellular processes among the genotypes (Fig. 6).

### Uniform protein accumulation profiles across three papaya genotypes

The analysis revealed a substantial disparity in the DAPs among the papaya genotypes, with the SS72/12 genotype exhibiting a significantly greater number of DAPs than the other genotypes at both the early and late stages of flower bud development. This discrepancy may be attributed to the distinct developmental stages of the flower buds sampled from each genotype. The selection of flower bud size as a sampling standard might have influenced the observed proteomic patterns, as developmental progression and bud sizes could vary across genotypes. Despite these differences, the analysis identified a subset of proteins that exhibited consistent accumulation patterns across all three studied genotypes (Table 2).

**Table 2:** Differentially accumulated proteins between hermaphrodite and female (XY^h^/XX) flower buds of three papaya genotypes at early and late developmental stages.

Pacid	Protein name	Arabidopsis orthologs	Peptide count	Score	Stage		Chr	Class functional
					Early	Late		
16,416,228	Hydroxymethylglutaryl-Coc synthase	HMGS	7	22.22	UP	–	4	Acyl-CoA metabolic process
16,420,853	ATP-citrate synthase alpha chain protein 2	ACLA-3	9	47.73	UP	–	3	Acyl-CoA metabolic process
16,422,019	Aspartate aminotransferase, mitochondrial	ASP1	14	55.37	–	UP	3	Amino acid metabolic process
16,423,471	3-dehydroquinate synthase, chloroplastic	AT5G66120	7	23.33	UP	–	4	Amino acid metabolic process
16,416,662	Glutamine synthetase nodule isozyme	GLN1-1	15	65.73	–	UP	9	Amino acid metabolic process
16,431,833	Pyridoxal 5'-phosphate synthase-like subunit PDX1.2	PDX1.2	9	33.89	UP	–	5	Amino acid metabolic process
16,421,959	DJ-1 protein homolog E-like	YLS5	2	6.57	Unique XY^h^	–	3	Auxin metabolic process
16,416,617	23.6 kda heat shock protein, mitochondrial isoform X2	HSP23.6	4	29.72	UP	–	9	Cadmium ion response
16,417,656	Alpha-L-arabinofuranosidase and beta-D-xylosidase	ASD1	5	8.53	–	UP	8	Carbohydrate metabolism
16,405,220	Alpha-L-arabinofuranosidase and beta-D-xylosidase	AT3G19620	19	38.24	–	UP	6	Carbohydrate metabolism
16,407,673	Putative alpha-mannosidase	AT3G26720	2	8.19	DOWN	–	–	Carbohydrate metabolism
16,411,511	Probable polygalacturonase	AT4G23500	6	19.67	UP	–	4	Carbohydrate metabolism
16,423,487	Glucan endo-1,3-beta-glucosidase 1	AT5G67460	4	20.05	DOWN	–	4	Carbohydrate metabolism
16,429,637	Myrosinase 5 OS = *Arabidopsis thaliana*	BGLU35	6	39.93	–	UP	3	Carbohydrate metabolism
16,404,021	Beta-glucosidase 40 OS = *Arabidopsis thaliana*	BGLU40	4	17.22	–	UP	2	Carbohydrate metabolism
16,416,524	Acidic endochitinase	CHIA	10	50.16	Unique XY^h^	–	9	Carbohydrate metabolism
16,423,395	Fructose-bisphosphate aldolase 1, chloroplastic	FBA2	15	51.52	–	UP	4	Carbohydrate metabolism
16,424,498	Fructose-bisphosphate aldolase 3, chloroplastic	PDE345	17	64.97	–	UP	2	Carbohydrate metabolism
16,426,415	Xyloglucan endotransglucosylase/hydrolase protein 2	XTH1	2	13.27	–	UP	3	Carbohydrate metabolism
16,420,647	UDP-D-apiose/UDP-D-xylose synthase 2	AXS2	13	48.28	UP	–	2	Carboxy-lyase activity
16,423,312	Soluble inorganic pyrophosphatase-like	PPa1	7	52.8	UP	–	4	Cell wall biogenesis
16,419,703	Probable pectinesterase/pectinesterase inhibitor 51	AT5G09760	5	15.11	UP	–	6	Cell wall modification
16,409,807	Xyloglucan endotransglucosylase/hydrolase 2	XTH16	4	15.9	UP	–	3	Cell wall polysaccharide metabolic process
16,409,474	Endoglucanase 24-like	GH9B18	5	16.8	Unique XY^h^	–	6	Cellulose catabolic process
16,411,521	Endoglucanase-like precursor	GH9B15	7	29.96	–	Unique XY^h^	–	Cellulose catabolic process
16,421,843	Cutin synthase	AT5G33370	2	8.99	–	Unique XY^h^	6	Cuticle development
16,418,378	Blue copper protein-like	AT1G72230	2	19.47	UP	–	1	Electron transfer activity
16,427,195	Early nodulin-like protein 1	ENODL3	3	3692	–	UP	3	Electron transfer activity
16,420,466	Blue copper protein-like	ENODL9	2	8.97	–	UP	7	Electron transfer activity
16,424,659	Acetyl-CoA carboxylase 1-like	ACC1	4	3.33	UP	–	1	Fatty acid biosynthetic process
16,419,976	GDP-L-fucose synthase	At1g17890	2	16.89	–	UP	6	GDP-L-fucose biosynthetic process
16,418,782	RAN GTPase-activating protein 1	RANGAP1	19	51.96	UP	–	–	GTPase activator activity
16,427,824	Phosphoglycolate phosphatase 1A	AT5G36790.1	5	21.95	DOWN	–	1	Hydrolase activity
16,421,839	GDSL esterase/lipase At5g33370-like	AT5G33370	14	67.48	UP	–	6	Hydrolase activity
16,415,240	GDSL esterase/lipase EXL3	AT5G42170	3	25.63	–	Unique XY^h^	1	Hydrolase activity
16,414,779	GDSL lipase/esterase	ESM1	5	23.32	DOWN	–	4	Hydrolase activity
16,419,252	Aldehyde oxidase GLOX-like	AT1G14430	6	20.25	–	Unique XY^h^	9	Integral component of membrane
16,404,669	Hydroxycinnamoyl-CoA: quinate/shikimate O-hydroxycinnamoyl transferase	HCT	5	27.97	–	UP	5	Lignin biosynthetic process
16,426,308	UDP-glucose 4-epimerase GEPI48	UGE5	8	34.96	UP	UP	4	Metabolic process of monosaccharides
16,410,983	Methionine S-methyltransferase	MMT	9	11.64	UP	UP	9	Metabolic process of S-adenosylmethionine
16,422,697	Mitochondrial uncoupling protein 1	PUMP1	2	10.82	UP	–	8	Mitochondrial transport
16,411,862	Cytosolic endo-beta-N-acetylglucosaminidase 1	ENGase85A	5	16.67	Unique XY^h^	–	2	Nitrogen compound metabolic process
16,415,424	Natterin-3 like	–	30	84.3	DOWN	–	6	No function
16,408,016	Fasciclin-like arabinogalactan protein 21	–	11	36.57	UP	–	4	No function
16,426,214	Leucine-rich repeat extensin-like protein 4	AT2G19780	7	39.62	–	UP	7	No function
16,413,335	Phospholipase A1-II gamma-like	At2g31100	3	12.08	–	Unique XY^h^	3	No function
16,405,261	Probable cinnamyl alcohol dehydrogenase 1	CAD5	15	63.06	–	UP	6	Phenylpropanoid biosynthesis
16,425,533	Probable cinnamyl alcohol dehydrogenase 9	CAD9	13	63.23	UP	–	–	Phenylpropanoid biosynthesis
16,416,437	Caffeic acid 3-O-methyltransferase	OMT1	10	49.32	UP	–	9	Phenylpropanoid biosynthesis
16,422,652	Peroxidase 17	AT2G22420	6	36.59	–	UP	4	Phenylpropanoid biosynthesis
16,428,257	Peroxidase 40	AT4G16270	14	54.35	UP	–	5	Phenylpropanoid biosynthesis
16,417,967	Putative Peroxidase 48	AT4G33870	3	13.95	Unique XY^h^	–	5	Phenylpropanoid biosynthesis
16,423,354	Peroxidase 72-like	AT5G66390	14	56.8	UP	Unique XY^h^	4	Phenylpropanoid biosynthesis
16,411,756	Soluble inorganic pyrophosphatase 4	PPa4	6	54.36	UP	UP	8	Phosphate-containing compound metabolic process
16,413,363	Probable cysteine protease RD21B	AT1G06260	20	68.48	–	UP	3	Protein catabolic process
16,405,685	Mesd domain-containing protein	AT2G46000	3	16.1	UP	–	5	Protein folding
16,404,975	T-complex protein 1 subunit eta	AT3G11830	16	47.6	UP	–	5	Protein folding
16,431,194	Berberine bridge enzyme-like 10	AT1G30720	10	38.33	–	Unique XYh	9	Proteolysis
16,418,608	Subtilisin-like protease SBT1.6	SBT1.6	33	69.74	UP	–	6	Proteolysis
16,422,477	Xylem cysteine peptidase 1	XCP1	9	52.38	–	UP	–	Proteolysis
16,429,914	Plastid-lipid-associated protein, chloroplastic	FIB	4	20.63	–	UP	7	Response to abscisic acid
16,414,902	2-alkenal reductase	AT5G16990	10	38.22	UP	–	5	Response to oxidative stress
16,425,735	glutamate–cysteine ligase, chloroplastic	GSH1	8	34.21	UP	–	4	Response to oxidative stress
16,424,279	Pathogenesis-related protein 5-like	TLP-3	2	15.51	–	UP	1	Response to stress
16,423,506	Serine/arginine-rich splicing factor SR45a	AT4G35785	5	15.28	–	DOWN	–	Splicing de RNA
16,414,345	Tetraketide alpha-pyrone reductase 1	TKPR1	2	14.04	Unique XY^h^	–	6	Sporopollenin biosynthesis
16,420,028	4-coumarate–coa ligase-like 1	ACOS5	18	58.79	–	Unique XY^h^	6	Sporopollenin biosynthesis
16,404,812	Type III polyketide synthase A-like	LAP6	10	48.33	–	Unique XY^h^	5	Sporopollenin biosynthesis

Chr: chromosome.

To ensure a comprehensive understanding of the molecular mechanisms underlying papaya flower development across different genotypes, our analysis focused on proteins exhibiting consistent accumulation patterns. Within this subset of proteins, the majority displayed as up-regulated or were unique to hermaphrodite samples. These proteins were found to be involved in essential pathways such as carbohydrate metabolism, cell wall constitution, phenylpropanoid biosynthesis, sporopollenin biosynthesis, and response to oxidative stress (Table 2).

**Figure 7:**
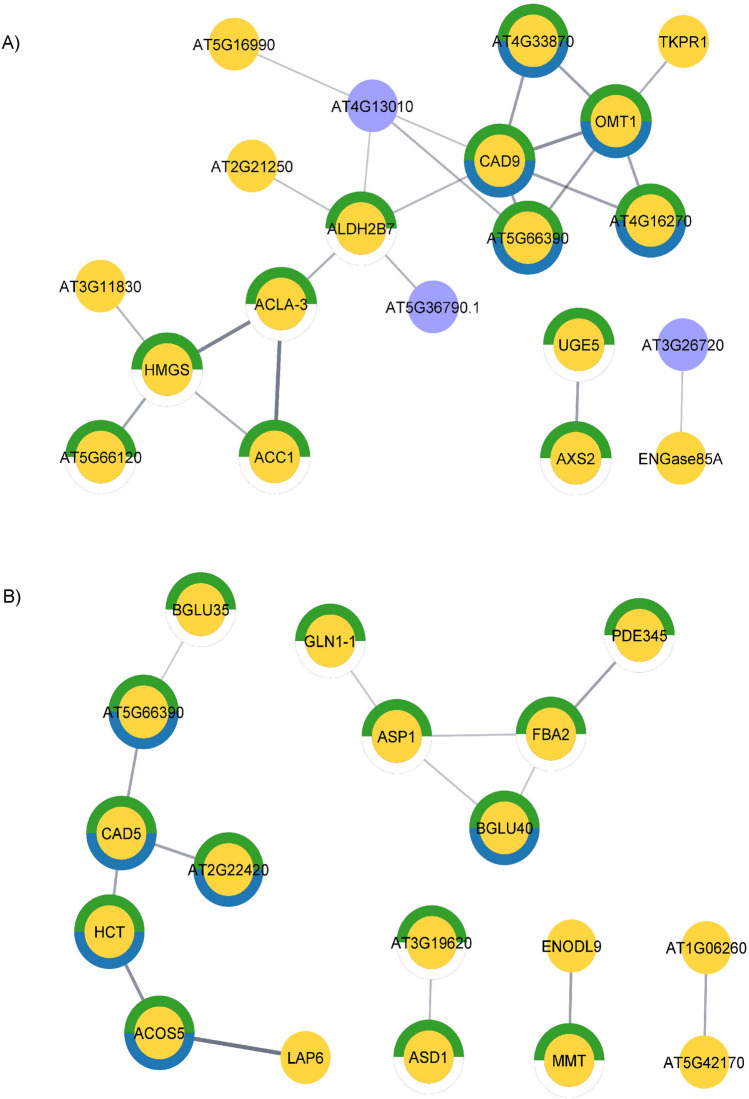
Predicted protein–protein interaction network in papaya flower buds at the early (**A**) and late (**B**) stages of development. Dashes represent interactions with a probability > 0.4. The yellow nodes represent proteins accumulated in hermaphrodite flowers. The blue nodes represent proteins accumulated in female flowers. The green circles indicate proteins involved in metabolic pathways, and the blue circles represent proteins associated with phenylpropanoid biosynthesis.

A protein–protein interaction (PPI) study was performed specifically with this subset of proteins, aiming to elucidate the functions of key proteins and their physical interactions within biological systems. This analysis revealed a greater number of predicted interactions among up-regulated proteins in hermaphrodite buds than among down-regulated proteins. Interestingly, few down-regulated proteins were predicted to be involved in the early stage of development, and no female proteins were detected at the final stage. Moreover, the interactions observed involved predominantly proteins associated with metabolic pathways and phenylpropanoid biosynthesis, emphasizing their significance in papaya flower development (Fig. 7).

## Discussion

Proteomic analysis revealed several differentially accumulated proteins (DAPs) in female (XX) and hermaphrodite (XY^h^) flower buds of contrasting genotypes at early and late stages of development. The time effect had a much greater influence on the protein profile than the sex effect, which indicates that the sex trait was attributed to a smaller set of genes.

The observed variations in protein accumulation among the papaya genotypes suggest that differential molecular processes underlie sexual differentiation. Notably, the greater number of DAPs in the SS72/12 genotype, particularly at the early stage of development, may indicate earlier gene activation related to sex determination in these plants. To focus on proteins potentially important for sexual differentiation across genotypes and developmental stages, the study specifically targeted those proteins with consistent accumulation patterns irrespective of genotype and time. This strategic approach allowed us to discern genotype-independent protein dynamics, facilitating the elucidation of core molecular processes driving flower development and differentiation.

The amount of DAPs was much greater in the SS72/12 genotype than in the other genotypes at both the early and late stages of development. The size of the flower buds was the standard used for sampling, which may have influenced the difference in the proteomic pattern, as both the development and the size of buds are different for each genotype. These results may indicate that gene activation was related to the sex of the SS72/12 plants earlier than to the other plants, as the other genotypes, mainly under early conditions, presented two times fewer DAPs between the sexes. This study focused only on proteins that exhibited the same accumulation pattern in the three studied genotypes due to differences in flower buds and with the aim of identifying proteins that play important roles in sexual differentiation regardless of genotype and time (Table 2).

Although transcriptome analyses focusing on the sexual differentiation of papaya have revealed several genes related to the biosynthesis of plant hormones and ABA, ROS, and auxin signaling pathways, most of the DAPs observed between the sexes in the present study were involved in carbohydrate metabolism, phenylpropanoid biosynthesis, and sporopollenin biosynthesis^13,32,33^. Furthermore, the genes encoding these proteins were all mapped to autosomal chromosomes or the autosomal region of chromosome 1. These discrepancies between transcriptomics and proteomics outcomes suggest that differences in post-transcriptional and post-translational regulation mechanisms could account for why certain genes identified in transcriptomics analyses may not correspond directly to the proteins identified in proteomics analyses. Additionally, variations in sample preparation, experimental conditions, and sensitivity of detection methods between transcriptomics and proteomics analyses could also contribute to differences in outcomes. In proteomics analyses, transcription factors are often not detected due to limitations in sensitivity detection compared to transcriptomics.

### Differences in carbohydrate metabolism between female and hermaphrodite flower buds in *Carica papaya*

A large group of DAPs in papaya flower buds were related to metabolism. Most of the DAPs were involved in carbohydrate and amino acid metabolism and were up-regulated at the early and late stages of hermaphrodite bud development. These findings reinforce previous studies indicating that energy metabolism plays a crucial role in sexual differentiation and flower bud development^34–36^.

Studies of dioecious plants have shown differences in the energy expenditure between male and female individuals. Male flowers of *Salix paraplexia* and *Silene latifolia* have greater reproductive costs due to greater biomass accumulation and energy consumption during reproduction^37^. In the present study, compared with female flowers, hermaphrodite flower buds at the early stage of development presented an increase in the accumulation of proteins related to carbohydrate metabolism. Proteins involved in monosaccharide and amino acid metabolism were up-regulated at the final stage of development.

Many of the cited pathways are not directly related to specific structures or functions of the androecium or gynoecium, as the proteins involved in these pathways are expressed in both sexes but at different levels. Thus, they can be classified as secondary sexual characteristics, which arose to increase sexual specialization after the evolution of dioecy and trioecy^38^.

In the present study, the ATP-citrate lyase A-3 (ACLA-3) and acetyl-CoA carboxylase 1 (ACC1) proteins, which play important roles in energy metabolism, were up-regulated in hermaphrodite flower buds at an early stage of development. The first protein is involved in the generation of acetyl-CoA, and the second protein is involved in the carboxylation of acetyl-CoA in plants; these two proteins are important steps in the synthesis of carbohydrates, amino acids, fatty acids, and flavonoids^39,40^.

The protein 3-hydroxy-3-methylglutaryl-coenzyme A synthase (HMGS), which was also up-regulated, interacted with the ACLA-3 and ACC1 proteins. HMGS is involved in the biosynthesis of phytosteroids, specifically the condensation of acetoacetyl-CoA and acetyl-CoA in the mevalonate (MVA) pathway. Informants with a mutation in the HMGS gene had infertile pollen grains, revealing that the tapetum requires the MVA pathway to develop tapetosomes and elaioplasts, which are required to form pollen coats^41^.

Most proteins enriched in carbohydrate metabolism in the present study play an important role in cell wall formation. Complex and highly dynamic plant cell walls are composed of interaction networks of polysaccharides, highly glycosylated proteins, and other polymers. This structure responds and adapts to normal processes of growth and development, as well as to biotic and abiotic stresses. Cellulose, hemicellulose, and pectins are the main carbohydrates of primary cell walls^42^.

The UDP-D-xylose synthetase (AXS2) protein and two probable proteins [pectin esterase/pectin esterase inhibitor (AT5G09760) and polygalacturonase (AT4G23500)] were up-regulated in papaya hermaphrodite flower buds at an early stage of development. The first two proteins are involved in synthesis^43,44^, and the latter is involved in pectin degradation^45^. Studies have shown that pectin is an essential factor for pollen development because it is the main constituent of primary cell walls^46,47^. Specifically, pectin is involved in the formation of the pollen grain wall, in addition to being a significant component of pollen carbohydrate reserves^48^.

UDP-D-glucose 4-epimerase (UGE5) is another protein identified in this work that is involved in pectin synthesis. This protein is involved in the synthesis of UDP-D-galactose, a nucleotide sugar precursor of several components of the cell wall matrix in addition to pectin, especially arabinogalactans, hemicellulose, and other polysaccharides and glycoproteins^49,50^.

Several extracellular glycosidic hydrolases play important but poorly understood roles in cell wall remodeling during plant growth^51^. In the present study, the enzyme xyloglucan endotransglucosylase/hydrolase 2 (XTH16) was up-regulated in papaya hermaphrodite flower buds at the early stage of development. The function of this enzyme is to promote cell wall plasticity through cleavage and reconnection of xyloglucan molecules, the main monosaccharide that makes up hemicellulose^52^. At least 33 genes have been reported in *Arabidopsis* to encode these enzymes^53^; the XTH3 homolog, expressed predominantly in *Arabidopsis* flower buds, seems to play a role in tapetum cell wall degradation^52^. Bifunctional alpha-L-arabinofuranosidase/beta-D-xylosidase (ASD1 and AT3G19620) is another glycosidic hydrolase that was DAP in hermaphrodite flower buds but was identified in buds at the final stage of development. This enzyme has already been identified in the roots and flowers of alfalfa (*Medicago sativa* L.) and has been characterized by the release of xylose and arabinose from the cell walls of these organs^51^.

The proteins glycosyl hydrolase 9B18 and 9B15 (GH9B18 and GH9B15, respectively) were unique to hermaphrodite flower buds at the early and late stages of development, respectively. Glycoside hydrolase family 9 (GH9) encodes a cellulase gene consisting of endo-β-1,4-glucanase, which is responsible for the process of cellulose synthesis and hydrolysis. Studies on the GH9 family of genes in plants have demonstrated their involvement in many plant development processes, such as cell elongation, anther dehiscence, pollen tube growth, abscission of branching nodes, and fruit ripening. More recently, GH9 genes characterized in wheat were shown to play important roles in anther development by targeting miRNAs and regulating cellulose levels via light and phytohormones^54^.

### Proteins involved in pollen development in *Carica papaya*

The outer wall of pollen and spores, called the exine, is highly resistant to chemical reagents and enzymes. The exine not only provides a protective barrier against pathogen attack, dehydration, and ultraviolet (UV) irradiation but also facilitates pollen recognition and stigma attachment^51^. Extensive evidence suggests that exine formation is a highly conserved polyketide biosynthetic pathway present in all land plant lineages and is likely associated with plant evolution. The tetracetide alpha-pyrone reductase 1 (TKPR1), acyl-CoA synthetase (ACOS5), and hydroxyalkyl alpha-pyrone synthase (LAP6) proteins were unique to papaya hermaphrodite flower buds; the first was identified in early-stage flower buds, and the last two were identified in the final stage of development. These enzymes have already been well characterized in several species, such as *A. thaliana*, rice, tobacco, and rapeseed, and are involved in the biosynthesis of sporopollenin, the main component of the exine^55,56^.

The composition of sporopollenin is not fully understood. Additionally, the exine is considered one of the most complex cell walls in plants. However, recent studies have shown that derivatives of the phenylpropanoid pathway are essential components of sporopollenin and act mainly in UV protection and maintenance of the genomic integrity of pollen^57^. The enrichment of pathways related to sporopollenin and phenylpropanoid biosynthesis in hermaphrodite samples across both developmental stages reaffirms their pivotal role in papaya flower development. Furthermore, the predicted interactions between proteins involved in these pathways suggest coordinated regulation and functional integration, highlighting their collective contribution to the synthesis and assembly of sporopollenin and exine components. This finding reinforces the notion that metabolic pathways associated with sporopollenin and phenylpropanoid biosynthesis are indispensable for ensuring the proper development and reproductive success of papaya flowers.

The phenylpropanoid pathway also provides intermediates for the synthesis of lignin, flavonoids, and hydroxycinnamoyl esters^58^. Furthermore, studies have shown that the fluorescence profiles of xylem lignin and pollen wall exines are similar. The protein caffeic acid 3-O-methyltransferase (OMT1), a probable cinnamyl alcohol dehydrogenase 9 (CAD9), and another probable cinnamyl alcohol dehydrogenase 1 (CAD5), which are already well characterized as having a key role in the synthesis of lignin, were up-regulated in the flower buds of hermaphrodites, the first two at the early stage and the latter at the late developmental stage. Plant O-methyltransferases (OMTs) constitute a large family of enzymes involved in the methylation of the oxygen atom of several secondary metabolites, including phenylpropanoids, flavonoids, and alkaloids^59^. The CAD5 protein plays a key role in the lignification of the anther endothelium. Moreover, plants harboring this gene exhibit anther dehiscence and pollen release failure, which causes male sterility^60^. Like the homologs mentioned above, CAD9 has been detected in the stems, leaves, and flowers of *Arabidopsis* plants; however, its biochemical functions are unknown, and CAD9 is highly expressed in male flower organs^61,62^.

Class III peroxidases (PRXs) are glycoproteins that play important roles in cell wall maturation and lignin formation. The following four PRXs were up-regulated in the present study: peroxidase 17 (AT2G22420), peroxidase 40 (AT4G16270), putative peroxidase 48 (AT4G33870), and peroxidase 72 (AT5G66390). In *Arabidopsis*, the PRX72 gene is expressed in stems, roots, leaves, and flowers, while the PRX17 gene is expressed in stems, flowers, and silica. The two corresponding proteins are located in the cell wall and are involved in lignin accumulation^63–65^. In the present study, PRX72 was up-regulated in hermaphrodite buds at the initial stage but only at the final stage of development, whereas PRX17 was up-regulated only at the final stage of development.

PRX40, which accumulates at an early stage of development, plays an important role in tapetum development and, consequently, a key role in male fertility. Because the tapetum is not considered lignified or suberized, extensins stand out as a potential substrate for PRX40^66^. Extensins are structural glycoproteins known to regulate cell size and shape^67^. Finally, putative peroxidase 48 was unique to hermaphrodite samples at an early stage of development. This protein is also expressed in the mature stems of *Arabidopsis*^68^. Despite the lack of evidence about its real function, this PRX was identified for the first time in floral organs in the present study.

Although the present study did not identify proteins linked to sex determination in papaya, the results contribute to the understanding of floral development in this crop. Most of the up-regulated proteins in hermaphrodite flower buds seem to be related to the development of male flower structures, especially during pollen wall formation. Importantly, protein genes involved in lignin synthesis, similar to those identified here, were highly expressed in hermaphrodite flowers compared to male flowers of *Lilium apertum*^69^.

Among the three sexes in which papaya plants are present, hermaphrodite plants are more vulnerable to abnormal flower production, while female plants are more stable in terms of flowering. The deformation of hermaphrodite flowers may represent an evolutionary strategy for overcoming environmental stress. The theory that carpelloid and pentandric flowers represent a trend of hermaphrodite plants returning to their female form has already been proposed^70^. The present study suggested that hermaphrodite flowers require greater energy expenditure during pollen development, specifically for the formation of their complex outer wall.

This study used for the first time the shotgun comparative proteomics method to analyze proteins from female and hermaphrodite flower buds of *C. papaya*, providing information about important molecular events in floral development in papaya. The carbohydrate metabolism, phenylpropanoid biosynthesis, and sporopollenin biosynthesis pathways were more enriched in hermaphrodite buds than in female buds at both stages of development. Most of these proteins play key roles in pectin, cellulose, and lignin synthesis. These compounds play important roles in cell wall formation and seem to be related to the development of male structures in flowers, especially in the formation of the pollen coat. The results suggest that hermaphrodite flowers require greater energy expenditure during development, probably due to the formation of complex external pollen walls.

### Supplementary Information

Supplementary Table S1.

Supplementary Table S2.

## Data Availability

The mass spectrometry proteomics data have been deposited to the ProteomeXchange Consortium via the PRIDE^71^ partner repository with the dataset identifier PXD050497. The list of all identified proteins is available in the supplementary material.
